# Prevalence and intensity of chronic pain and self-perceived health among elderly people: a population-based study^1^

**DOI:** 10.1590/0104-1169.3591.2465

**Published:** 2014

**Authors:** Lilian Varanda Pereira, Patrícia Pereira de Vasconcelos, Layz Alves Ferreira Souza, Gilberto de Araújo Pereira, Adélia Yaeko Kyosen Nakatani, Maria Márcia Bachion

**Affiliations:** 2 PhD, Adjunct Professor, Faculdade de Enfermagem, Universidade Federal de Goiás, Goiânia, GO, Brazil; 3 MSc, RN; 4 MSc, RN, Hospital das Clínicas, Universidade Federal de Goiás, Goiânia, GO, Brazil. RN, Secretaria Municipal de Saúde, Prefeitura de Goiânia, Goiânia, GO, Brazil; 5 PhD, Adjunct Professor, Departamento de Enfermagem em Educação e Saúde Comunitária, Universidade Federal do Triângulo Mineiro, Uberaba, MG, Brazil; 6 PhD, Associate Professor, Faculdade de Enfermagem, Universidade Federal de Goiás, Goiânia, GO, Brazil; 7 PhD, Full Professor, Faculdade de Enfermagem, Universidade Federal de Goiás, Goiânia, GO, Brazil

**Keywords:** Aged, Pain Measurement, Chronic Pain, Self-Assessment

## Abstract

**OBJECTIVE:**

to identify the prevalence and intensity of chronic pain among elderly people of the community and to analyze associations with the self-perceived health status.

**METHOD:**

cross-sectional study with a populational sample (n=934), conducted through household interviews in the city of Goiânia, Brazil. The intensity of chronic pain (existing for 6 months or more) was measured using a numerical scale (0-10) and the self-perceived health through a verbal scale (very good, good, fair, poor, very poor). For the statistical analysis, the absolute frequency and percentage, CI (95%), Chi-square test, Odds ratio, and regression analysis were used. Significance of 5%.

**RESULTS:**

The prevalence of chronic pain was 52.8% [CI (95%):49.4-56.1]; most frequently located in the lower limbs (34.5%) and lumbar region (29.5%); with high or the worst possible intensity for 54.6% of the elderly people. The occurrence of chronic pain was associated with (p<0.0001) a worse self-perception of health (OR=4.2:2.5-7.0), a greater number of chronic diseases (OR=1.8:1.2-2.7), joint disease (OR=3.5:2.4-5.1) and the female gender (OR=2.3:1.7-3.0). A lower intensity of chronic pain was associated with a better self-perception of health (p<0.0001).

**CONCLUSION:**

the majority of the elderly people of the community reported chronic pain, of a severe intensity, and located in areas related to movement activities, thus influencing the morbidity and mortality of this population.

## Introduction

Aging of the global population has been accompanied by a rising prevalence of chronic and degenerative diseases^(^ 1 ^)^ and, consequently, a higher incidence of pain and disability^(^ 2 ^-^ 3 ^)^. National and international studies show that the prevalence of chronic pain among elderly people of the community ranges from 29.7% to 89.9%^(^ 4 ^-^ 8 ^)^. Often located in the upper and lower limbs, the back (lumbar region), neck and joints, the face, abdomen, knee, hip, chest and rectum^(^ 4 ^-^ 5 ^,^ 8 ^-^ 9 ^)^, this pain has been reported with moderate/severe intensity^(^ 4 ^-^ 5 ^)^. In the elderly population, a relationship has been found between the occurrence of chronic pain^(^ 10 ^)^, its increased intensity, and a prolonged period of living with it^(^ 11 ^)^ and a worse self-perception of the health status, corroborating the findings of a study conducted in Canada^(^ 12 ^)^, in which elderly people reported a worse self-perception of health in the presence of pain. However, studies focusing on this topic are scarce, especially related to the Brazilian aging scenario. Furthermore, the study of the factors that influence the health conditions in the elderly population, such as chronic pain, may indicate intervention strategies and action planning that promote well-being and, additionally, allow the impact of the interventions on the health and quality of life of elderly people to be evaluated.

The aim of this study was to estimate the prevalence and intensity of chronic pain and to analyze associations between these variables and the self-perceived health status among elderly people of the community.

## Method

This cross-sectional type, population-based study, with an epidemiological survey design, was carried out by the Health Surveillance Network for Elderly People (REVISI), in 2010 in Goiânia, Goiás State, Brazil.

The minimum required sample of the elderly population (persons 60 years of age or older, the age established for the elderly person in Brazil, according to Law No. 8.842 of January 4, 1996) of Goiania (7% of the total population; 1,249,645 - base year 2007) was calculated based on an estimated prevalence of pain in the population, with a confidence level of 95%, expected prevalence of 30%, absolute precision of 5 percentage points, DEEF of 1.8, and an increase of 11% for losses. The representative sample of the elderly population living in the urban zone of the city of Goiânia consisted of 934 individuals.

The concept of living at home was "sleeping in the residence for more than four days per week". Those who lived at home, however, were not found after three attempted visits by the observer were excluded, as were those who achieved scores <13 in the evaluation of cognitive ability through the Mini Mental State Examination (MMSE)^(^ 13 ^)^, which provided a total of 872 elderly people.

The geographical area of the study was defined based on census sectors (CS) and, for the demarcation of these sectors, field maps and data-sheets provided by the Brazilian Institute of Geography and Statistics (IBGE) were used. The basic digital city map of Goiânia (MUBDG), completed in 1996, was supplied by COMDATA - municipal institution responsible for constructing the digital grid of the city. This map was used in the digitization of the 1,068 census sectors. Of this total, 912 strictly urban CS, were used for the localization of the elderly people. 

The mean population per SC was 980 individuals and, considering that elderly people composed 7% of the population, there was a mean of 16.3 elderly people per SC, with the 56 required SC randomly selected from among the 912. The selection was made by means of a random number table created using an electronic randomization system. Of the 934 elderly people of the random sample, 9 (nine) were excluded due to achieving scores <13 in the MMSE, and 53 were considered as losses because they needed help completing the questionnaire or because they had not completed the questions related to the pain evaluation. Thus, 872 elderly people comprised the study sample.

For each CS, the first block was selected and, within this, a corner where the data collection was initiated. From the selected corner, the first residence was visited, excluding any property that was not residential. For each SC selected, and from the block defined, the households were visited until 17 elderly people had been interviewed.

Data were collected from the elderly people in their homes by properly trained people. After identification by the interviewer, the elderly people were informed about the study aims, research methods, risks/benefits, guarantee of anonymity and confidentiality of the data. The elderly people who met the inclusion criteria were invited to participate and those who agreed were given two copies of the Terms of Free Prior Informed Consent. Next, the questionnaire, composed of 12 sections (identification, social profile, caregiver, general health and family history, with a arterial pressure check and recording of the weight and height, habits of life, pain evaluation, respiratory symptoms, functional evaluation, quality of life, frailty, falls, access to healthcare services and the Mini Mental State Examination), was applied.

The outcome variable for this study was chronic pain (existing for six months or more^(^ 14 ^))^. Measured from the main pain, i.e., the one that most bothered the elderly person, felt in the seven days preceding the interview. To measure the intensity, a numerical range (0-10) was used, in which zero=no pain; 1,2,3 and 4=mild pain; 5 and 6=moderate pain; 7,8 and 9=severe pain and 10=worst possible pain. Exposure variables: self-perceived health status (evaluated using a verbal rating scale ("very good", "good", "fair", "poor", "very poor"; gender (male/female) and age by age group (60-69 years=young elderly; 70-79=elderly, 80 years and over=very elderly), and number and type of self-reported chronic diseases.

Other variables, such as marital status (married, single, widowed and divorced); education (illiterate, elementary education, high school education, higher education); employment status (working, not working), and living alone (yes/no), were collected to characterize the sample.

The REVISI project was approved by the Research Ethics Committee of the Federal University of Goiás, protocol No. 050/2009, and funded by the Foundation for Research Support of the State of Goiás - No. 001/2007. All the elderly people of this study signed the Terms of Free Prior Informed Consent.

The data were analyzed through absolute frequencies and percentages. The associations between the variables of interest were evaluated from the chi-square test, Odds Ratio and regression analysis. The significance level for all tests was 5%.

## Results

Among the 872 elderly people that participated in this study, there was a prevalence of women (62.3%), aged between 60 and 69 years (50.1%), married (50.3%), with an elementary education level (48.6%), living with another person/people (86.8%), and unemployed (79.8%).

Regarding the clinical characteristics, 45.4% of the elderly people reported "very good" or "good" health and 10.4% "poor" or "very poor" health. Two or more diseases were mentioned by 50.2%; the presence of hypertension was reported by 73.8%.

The prevalence of chronic pain was 52.8% [CI (95%): 49.4%-56.1%]. Among the individuals with chronic pain (n=460), 49.6% reported feeling it in a single location and 15.1% in more than three locations (Table 2). The pain was more frequently located in the lower limbs (34.5%), lower back (29.5%), head/face/neck (16.2%) and shoulder/upper limbs (10.0%). Regarding the intensity, 42.1% reported severe pain, 25.9% moderate, 19.4% mild and 12.6% considered that they felt the worst pain possible.

**Table 1:** Socioeconomic and demographic characteristics of the elderly people Goiânia, GO, Brazil, 2010

Variables		Elderly people	
		n	%
Gender (872)			
	Male	329	37.7
	Female	543	62.3
Age (872)			
	60-69 years	437	50.1
	70-79 years	286	32.8
	80 years or over	149	17.1
Martial status (867)			
	Married	436	50.3
	Single	80	9.2
	Widowed	271	31.3
	Separated	80	9.2
Education (869)			
	Illiterate	168	19.3
	Elementary	422	48.6
	High school	197	22.7
	Higher	82	9.4
Lives alone			
	Yes	114	13.2
	No	752	86.8
Works			
	Yes	172	20.2
	No	680	79.8

**Table 2:** Clinical characteristics of the elderly people. Goiânia, GO, Brazil, 2010

Variables		Elderly people	
		n	%
Self-perceived health (n=837)			
	Very good/Good	380	45.4
	Fair	370	44.2
	Poor/Very poor	87	10.4
Number of chronic diseases (n=872)			
	None	162	18.6
	One	272	31.2
	Two or more	438	50.2
Chronic diseases (n=710)*			
	Arterial hypertension	524	73.8
	Joint disease	189	26.6
	Diabetes	161	22.7
	COPD	129	18.2
	AMI	51	7.2
Chronic Pain (n=872)			
	Yes	460	52.8
	No	412	47.2
Locations of the pain (n=460)^†^			
	One	228	49.6
	Two	88	19.1
	Three	70	15.2
	Four or more	74	16.1
Intensity of the pain (n=428)^‡^			
	Mild	83	19.4
	Moderate	111	25.9
	Severe	180	42.1
	Worst possible	54	12.6

* Multiple response, percentage calculated for those who reported having one or more chronic diseases

† Percentage calculated for those who reported feeling chronic pain

‡ 32 elderly people with chronic pain did not report the pain intensity due to not feeling it the previous 7 days

The presence of chronic pain was significantly associated with a poorer self-perception of the health status (χ^2^=46.9, p<0.0001), a greater number of existing chronic diseases (χ^2^=51.4, p<0.0001), the presence of joint disease (χ^2^=51.6, p<0.0001), and the female gender (χ^2^=33.9, p<0.0001). Regarding the self-perceived health status, those who perceived their health negatively ("poor or very poor") were more likely to report chronic pain than those with "very good or good" self-perceived health (OR=4.2; 2.5-7.0), as shown in Table 3.

**Table 3:** Clinical characteristics according to reports of chronic pain. Goiânia, GO, Brazil, 2010

Characteristics		Chronic pain								χ^2^	p
		Yes			No			Odds Ratio			
		n	%		n	%		OR	CI(95%)		
Self-perceived health* (n=837)										46.9	<0.0001
	Very good/Good	158	41.6		222	58.4		1.0	-		
	Fair	226	61.1		144	38.9		2.2	1.9-3.0		
	Poor/Very poor	65	74.7		22	25.3		4.2	2.5-7.0		
Number of chronic diseases (n=872)										51.4	<0.0001
	None	53	32.7		109	67.3		1.0			
	One	128	47.1		144	52.9		1.8	1.2-2.7		
	Two or more	279	63.7		159	36.3		4.2	2.9-6.2		
Joint disease (n=854)										51.6	<0.0001
	No	307	46.2		358	53.8		1.0			
	Yes	142	75.1		47	24.9		3.5	2.4-5.1		
Gender (n=872)										33.9	<0.0001
	Male	132	40.1		197	59.9		1.0			
	Female	328	60.4		215	39.6		2.3	1.7-3.0		

* Eleven (11) subjects did not respond regarding self-perceived health and joint disease

Concerning the number of chronic diseases, to have reported one disease almost doubled the chance of a positive report regarding chronic pain (OR=1.8; 1.2-2.7). This chance was three times greater for the elderly people who reported joint disease (OR=3.5; 2.4-5.1). In addition, considering gender, being female doubled the chance of reporting chronic pain (OR=2.3; 1.7-3.0) (Table 3). 

The separate analysis for men and women of the relationship between self-perceived health status and chronic pain, showed that, among the women (n=319) 45.8% (97\212), 68.4% (167\244), and 83.3% (55\66) perceived their health as "very good/good", "fair" and "poor/very poor", respectively. Among the men (n=130), these proportions were 36.3% (n=61\168), 46.8% (n=59\126) and 47.6% (n=10\21), respectively. Self-rated health status and chronic pain were significantly associated with the female gender (χ^2^=41.6; p<0.0001) following the general analysis; in males, however, this association was not significant (χ^2^=3.7; p=0.1604).

In the women, the chance of evaluating the health as "fair" was 2.6 times higher compared with the evaluation "very good or good" (OR=2,6;1.7-3.8); and nearly six times higher compared to the evaluation of "poor and very poor" health (OR=5.9;2.9-12.0). In the men, these chances were lower (OR=1.5;0.9-2.5 for "fair" and 1.6;0.6-3.9 for "poor and very poor").

In Table 4, it can be observed that self-perceived health was associated with a greater intensity of chronic pain (χ^2^=72.9; p<0.0001).

**Table 4:** Self-perceived health according to the intensity of chronic pain. Goiânia, GO, Brazil, 2010

Self-perceived health	Intensity of the Pain (n=419)*										
	Mild			Moderate			Severe			Worst possible	
	n	%		n	%		n	%		n	%
Very good/Good	49	34.0		44	39.6		45	31.3		6	4.2
Fair	30	13.6		56	26.1		99	46.1		30	14.0
Poor/Very poor	3	5.0		9	15.0		31	51.7		17	28.3

* 41 elderly people with chronic pain did not report pain in the previous 7 days; ?2=56.5; p<0.0001

**Figure 1:**
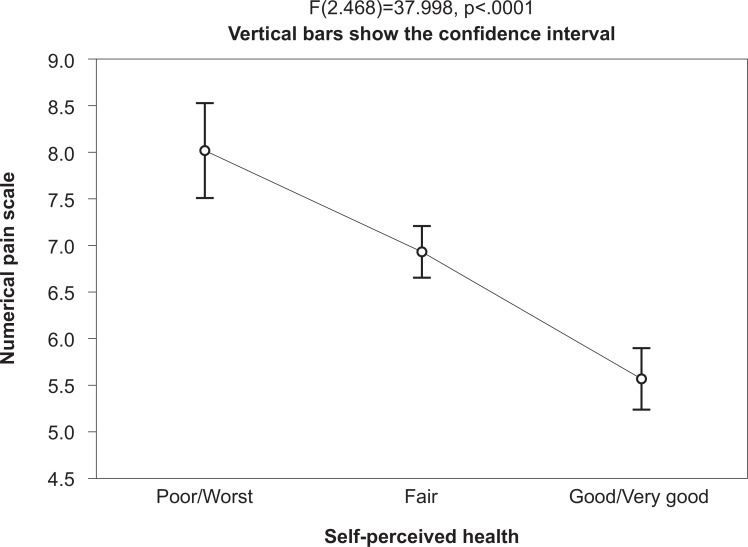
Numerical pain scale (0-10) in relation to the self-perceived health (Poor/Worst x Fair: p=0.008; Poor/Worst x Very good/Good: p

Also related to the intensity of chronic pain and self-perceived health, the regression analysis (Figure 1) showed that, according to the numerical scale (0-10), when the pain scores were lower, indicating less severe pain, the elderly people evaluated themselves as having better health.

## Discussion

Studies^(^ 4 ^-^ 9 ^,^ 15 ^)^ have shown that the prevalence of chronic pain can range from 29.7% to 89.9%. This variation may result from the influence of factors that include the definition of the time established for the pain to be classification as chronic, regional sociodemographic differences, the methodology used for data collection and variations among the data collection instruments used^(^ 16 ^)^.

Given the extent of the chronic pain problem, the findings of the present study corroborate those of national and international studies, indicating a high proportion of pain complaints among the elderly people of the community, which may mean prolonged suffering, which is sometimes ignored and under-treated. Cross-sectional studies^(^ 4 ^-^ 5 ^)^, performed in Brazil with populational samples, conducted through household interviews and temporal characterization of the chronic pain as existing for six months or more, showed a similar prevalence of chronic pain (51.7%) to the present study among 451 elderly people of Londrina, PR, and a lower prevalence (29.7%) among 1,271 elderly people of the city of São Paulo, SP. Accordingly, the need is highlighted for further research with representative samples of the elderly population, in other regions of Brazil, with a view to identifying the extent of chronic pain prevalence in this age group and regional factors that may protect or expose the elderly people to the persistent painful experience. The greater representativity for the women corroborates the findings of studies conducted with the elderly population^(^ 4 ^,^ 15 ^-^ 16 ^)^, indicating the feminization of old age.

The intensity of the chronic pain was high (*severe* and *worst possible pain* for 54.6% of the elderly people), as also shown in other studies^(^ 4 ^,^ 15 ^-^ 16 ^)^, in which the proportions of severe pain reached over 50.0%. In Spain^(^ 17 ^)^, the prevalence of moderate to sever intensity pain was 86.4%; In Brazil^(^ 4 ^)^, this reached 45.8% for moderate pain and 46.0% for severe and very severe pain and, among these subjects, half reported daily pain.

Although the majority of the measuring instruments used in the different studies have ordinal level type scales, the categories often presented during the judgments varied, complicating comparisons. The literature indicates a lack of standardization in the use of scales to measure the intensity of pain and, even when they are similar, as in the case of the numerical scale (0-10), often the ranking of the scores that characterize the intensity as "mild", "moderate", "severe" and "worst possible pain" is not the same. This fact adds limitation to the subjective measures of pain, as mild, moderate and severe pain are similar categories for the different populational groups^(^ 16 ^)^.

A moderate, severe and very severe pain intensity was mentioned by many of the elderly people who reported pain^(^ 4 ^,^ 15 ^)^, indicating the importance of the measurement in the overall evaluation process of the elderly person. Severe pain tends to be more disabling, affecting the quality of life, reducing social contact and increasing impairments in relationships and leisure activities.

As found in the present study, there are reports of the daily existence with chronic pain, including among those with pain in the lower limbs and back, locations directly related to movement activities^(^ 4 ^-^ 6 ^,^ 9 ^,^ 18 ^)^. Chronic diseases cause pain that may be reported in locations that interfere with the autonomy and independence, imposing functional limitations. A cross-sectional study with an elderly population^(^ 8 ^)^ reveled that some pain locations decline with advancing age (head) and others become more frequent (feet, hips and knees). A literature review^(^ 19 ^)^ found the presence of lumbar pain with advancing age, especially in women. In Minas Gerais^(^ 20 ^)^, the authors found a high incidence of pain in the supportive joints, suggestive of rheumatic afflictions of the osteo-degenerative type, considered to be the main causes of pain in the population. It should be noted that with the emergence of bodily failure in old age, specifically in the musculoskeletal system, the aging process contributes to the emergence of chronic-degenerative diseases and, in this case, the lower limbs are included in a high percentage of the described clinical conditions^(^ 8 ^)^.

Pain can be considered one of the most uncomfortable and desperate situations that affects people and constitutes an important issue in the lives of elderly people^(^ 21 ^)^. In Toronto, Canada, high intensity pain for an extended period was associated with worse self-perceived health^(^ 11 ^)^, as also observed in this study. Similarly, in Sao Paulo, Brazil^(^ 22 ^)^, pain was significantly associated with a poorer self-perception of health. In Finland^(^ 23 ^)^, a study presented a strong association between self-perceived health and chronic pain, regardless of chronic diseases, gender or age.

There was a significant association between self-perceived health and chronic pain among the women of this study, however, not among the men. A study indicated that men give a worse evaluation of their health when they are at greater risk of being affected by a fatal event^(^ 24 ^)^. It is assumed that, due to the life expectancy of women being higher than that of men, they have an increased chance of developing chronic and degenerative diseases and pain and therefore evaluate their health more negatively, due to associating it with losses in the quality of life.

In Canada, prolonged high intensity pain was associated with worse self-perceived health^(^ 11 ^)^, as in the present study. In Finland^(^ 23 ^)^, the prevalence of fair health was 38.1% among those who reported pain once a week (for 46.0% the pain was daily or continuous), and the prevalence of poor health was 5.1% among those who reported pain once a week (for 31.7% the pain was daily or continuous). The proportions increased with the higher frequency of reports of more intense pain.

To achieve pain control is a right of the general population and also the elderly person^(^ 4 ^,^ 11 ^,^ 21 ^)^. The constant pain complaints, associated with a worse self-perception of health, highlight the need for longitudinal studies that investigate the influence of persistent pain on mortality and morbidity in elderly people. In this sense, it is essential that chronic pain is routinely evaluated and measured in the healthcare services and that the entire team is trained to intervene or to refer people for specialist treatment^(^ 25 ^)^. For this, health professionals should receive adequate training, from the undergraduate course, to conduct an evaluation of the occurrence of pain in the general population, across the life cycle, and within their competences, so that they prepared to intervene, using the diversity of approaches available to work in an interdisciplinary manner.

A limitation of the study was being part of a larger study, in which several outcomes were investigated and not specifically chronic pain, which did not allow a deeper investigation of this axis in the original investigation. However, the data obtained from a representative sample of the elderly population of the Brazilian metropolis investigated represent an important contribution, representing the situation of the elderly population of the community faced with the persistent painful experience. A further limitation of the study is that only elderly individuals with MMSE scores >13, and who were able to hear and speak were evaluated, which excluded individuals with severe dementia, severe deafness and language deficits, limiting the generalizability of the results to this populational group.

## Conclusion

The majority (52.7%) of the elderly people suffered chronic pain, of high intensity (54.6%), in locations (lower limbs, lumbar region, head/face/neck, shoulders/upper limbs) that can compromise movement activities, and other functional activities of daily living, imposing disability and losses in the quality of life. This implies a revision of the paradigms of the approach to chronic noncommunicable diseases, beyond the control of the disease or its treatment itself, with pain management by the multidisciplinary team. Furthermore, the impact of the pain on treatment adherence must be evaluated, since the painful occurrence can interfere with the performance of physical activities and exercise, generally recommended for the control of diseases such as diabetes, hypertension, obesity, and dyslipidemia, among others.

The association between the occurrence of chronic pain and perceptions of poor or very poor health among the elderly people, together with the association between lower pain intensity scores with better perceived health, indicate the importance of including, in the overall assessment of the elderly person, the measurement of the painful experience, seeking adequate maintenance, replacement or supplementation of the analgesic therapy and greater impact in reducing morbidity and mortality in this population.
